# Segmentation of pre- and posttreatment diffuse glioma tissue subregions including resection cavities

**DOI:** 10.1093/noajnl/vdae140

**Published:** 2024-08-16

**Authors:** Saif Baig, Igor Vidic, George M Mastorakos, Robert X Smith, Nathan White, Suzie Bash, Anders M Dale, Carrie R McDonald, Thomas Beaumont, Tyler M Seibert, Jona Hattangadi-Gluth, Santosh Kesari, Nikdokht Farid, Jeffrey D Rudie

**Affiliations:** Department of Radiology, Nassau University Medical Center, East Meadow, New York, USA; Cortechs.ai, San Diego, California, USA; Cortechs.ai, San Diego, California, USA; Cortechs.ai, San Diego, California, USA; Cortechs.ai, San Diego, California, USA; Radnet, San Fernando Valley, California, USA; Department of Radiology, University of California, San Diego, San Diego, California, USA; Department of Radiology, University of California, San Diego, San Diego, California, USA; Department of Radiology, University of California, San Diego, San Diego, California, USA; Department of Radiology, University of California, San Diego, San Diego, California, USA; Department of Radiation Medicine and Applied Sciences, University of California, San Diego, San Diego, California, USA; Department of Bioengineering, University of California, San Diego, San Diego, California, USA; Department of Radiation Medicine and Applied Sciences, University of California, San Diego, San Diego, California, USA; Pacific Neuroscience Institute, Santa Monica, California, USA; Department of Radiology, University of California, San Diego, San Diego, California, USA; Department of Radiology, University of California, San Diego, San Diego, California, USA; Department of Radiology, Scripps Clinic, La Jolla, California, USA

**Keywords:** artificial intelligence, brain tumors, neuro-oncology, neural networks, segmentation

## Abstract

**Background:**

Evaluating longitudinal changes in gliomas is a time-intensive process with significant interrater variability. Automated segmentation could reduce interrater variability and increase workflow efficiency for assessment of treatment response. We sought to evaluate whether neural networks would be comparable to expert assessment of pre- and posttreatment diffuse gliomas tissue subregions including resection cavities.

**Methods:**

A retrospective cohort of 647 MRIs of patients with diffuse gliomas (average 55.1 years; 29%/36%/34% female/male/unknown; 396 pretreatment and 251 posttreatment, median 237 days post-surgery) from 7 publicly available repositories in The Cancer Imaging Archive were split into training (536) and test/generalization (111) samples. T1, T1-post-contrast, T2, and FLAIR images were used as inputs into a 3D nnU-Net to predict 3 tumor subregions and resection cavities. We evaluated the performance of networks trained on pretreatment training cases (Pre-Rx network), posttreatment training cases (Post-Rx network), and both pre- and posttreatment cases (Combined networks).

**Results:**

Segmentation performance was as good as or better than interrater reliability with median dice scores for main tumor subregions ranging from 0.82 to 0.94 and strong correlations between manually segmented and predicted total lesion volumes (0.94 < *R*^2^ values < 0.98). The Combined network performed similarly to the Pre-Rx network on pretreatment cases and the Post-Rx network on posttreatment cases with fewer false positive resection cavities (7% vs 59%).

**Conclusions:**

Neural networks that accurately segment pre- and posttreatment diffuse gliomas have the potential to improve response assessment in clinical trials and reduce provider burden and errors in measurement.

**Key Points**

- Neural networks accurately segmented pre- and posttreatment glioma tissue subregions.
- A network trained on pre- and posttreatment glioma cases performed better than dedicated networks.
- Neural networks performed as good or better than interrater reliability.

**Importance of the Study**

While there are many prior studies on brain tumor segmentation, this is one of a handful of studies to evaluate posttreatment high and low gliomas and the only one that also distinctly segments resection cavities. There is significant potential for this work to be applied to glioma treatment response assessment in daily clinical practice and clinical trials in order to reduce errors in measurement and provider burden.

Multiparametric MRI (mpMRI) is the standard of care for evaluating disease progression and treatment response in patients with diffuse glioma. Artificial intelligence methods have shown significant promise in automated delineation and quantification of diffuse glioma tumor subregions. Fast and accurate segmentation of diffuse glioma tissue volumes could reduce interrater variability and increase workflow efficiency for both treatment planning and routine longitudinal radiographic assessment.

Convolutional neural networks have excelled in the task of biomedical segmentation with performance reaching human interrater reliability. Advances in the field of glioma segmentation have been supported by data made available through The Cancer Imaging Archive (TCIA)^1^ as well as the yearly multimodal brain tumor segmentation challenge (BraTS).^2,3^ The BraTS curated data set of preoperative brain MRIs with expert segmentations of tumor subregions has propelled the development of advanced segmentation and prognostication algorithms. In particular, the nnU-Net,^4^ a self-configuring segmentation network, has won multiple recent BraTS.^5,6^

The most common indication for glioma imaging is disease burden assessment after treatment with maximal safe resection, radiation, and chemotherapy. Unfortunately, the BraTS data set is limited to preoperative/pretreatment MRIs. The varied appearance of the posttreatment brain, which includes resection cavities, gliosis, and radiation changes adds further challenges in the development of a clinically useful tool. Limited prior studies have evaluated segmentation of posttreatment diffuse gliomas^7–12^ or resection cavities.^13,14^ Contouring of resection cavities is important for patients receiving adjuvant radiotherapy after surgical resection. Additionally, given that necrotic core and resection cavities can appear similar, distinguishing them would be ideal for important for evaluation total tumor volumes. A tool that could accurately segment both pre- and posttreatment diffuse glioma tumor subregions, including resection cavities, would be useful in daily clinical radiology practice for assessing treatment response, clinical trials, and in guiding adjuvant radiation therapy, which is a laborious and time-intensive manual process.

We sought to develop an algorithm that could accurately segment glioma tissue subregions including resection cavities in glioma patients that were both pre- and posttreatment. Furthermore, we wanted to evaluate whether separate networks dedicated to pretreatment or posttreatment gliomas would be better or equivalent to a network trained on gliomas regardless of prior treatment history.

## Materials and Methods

### Data

A retrospective sample consisting of 647 mpMRI from patients with diffuse gliomas (55 ± 13 SD years; 29% female, 36% male, 34% unknown; 87% (563) high grade gliomas) were included. Posttreatment patients had a median of 237 days from surgery (range 0–1368) and most had undergone adjuvant chemoradiation. Complete demographic information can be found in Table 1. The sample contained 355 annotated pretreatment scans from BraTS 2020 with 11 scans excluded for missing/incomplete images and 3 scans excluded for poor image quality from the original 369 scans available in BraTS 2020. An additional 41 pretreatment and 251 posttreatment scans were used from 7 other publicly available TCIA repositories with permission (ACRIN-DSC-MR-Brain, ACRIN-FMISO-Brain, CPTAC-GBM, Ivy-GAP, brain tumor progression, TCGA-LGG, and TCGA-GBM), with patients selected across a range of posttreatment time intervals and excluding scans with incorrect body parts (*n* = 164), missing sequences (*n* = 344), or artifacts (*n* = 47). Overall, the data were acquired from at least 59 different sites and 26 unique scanner models. This data set and training/test/generalization split represents the exact data and data split for the FDA-cleared commercial product Neuroquant Glioma, Cortechs.ai (San Diego), but was trained separately.

**Table 1.** Patient Demographics and Tumor Volumetric Information

	Training/Validation	Test	Generalization	Total
Demographics				
Number of Patients	536	67	44	647
Age (y)	54.8 (±13.1)	59.5 (±13.9)	53.4 (±14.5)	55.1 (±13.4)
Male	190 (35%)	20 (30%)	25 (57%)	235 (36%)
Female	150 (28%)	20 (30%)	19 (43%)	189 (29%)
Unknown	196 (37%)	27 (40%)	0 (0%)	223 (34%)
Operation status				
Pre-Op	310 (57%)	42 (63%)	44 (100%)	396 (61%)
Post-Op	226 (42%)	25 (37%)	0 (0%)	251 (39%)
Primary cancer types				
High grade	476 (88%)	62 (93%)	25 (57%)	563 (87%)
Low grade	60 (11%)	5 (7.5%)	19 (43%)	85 (13%)
Tumor volumetric information				
Presence of ET	478 (89%)	59 (85%)	29 (62%)	566 (87%)
Presence of RC	176 (33%)	20 (28%)	0 (0%)	196 (30%)
Presence of NCR	366 (68%)	43 (62%)	35 (78%)	444 (69%)
WL volume (cm^3^)	96.4 (±60.4)	87.9 (±60.0)	94.8 (±65.7)	95.4 (±60.7)
SNFH volume (cm^3^)	63.7 (±4.4)	58.3 (±42.7)	53.7 (±38.4)	62.4 (±43.6)
TC volume (cm^3^)	32.7 (±32.7)	29.7 (±31.4)	41.0 (±36.5)	35.0 (±32.9)
ET volume (cm^3^)	21.7 (±20.4)	17.0 (±16.9)	19.4 (±22.6)	21.8 (±20.2)
NCR volume (cm^3^)	19.4 (±27.1)	12.7 (±21.6)	21.7 (±30.6)	20.0 (±27.3)
RC volume (cm^3^)	18.9 (±22.1)	7.1 (±17.2)	0 (±0)	19.4 (±22.3)

ET = enhancing tissue; NCR = necrotic core; RC = resection cavity; SNFH = surrounding nonenhancing FLAIR hyperintensity. The whole lesion (WL) extent is defined as the union of all 3 distinct subregions (ET, SNFH, and NCR), excluding resection cavity. Tumor core (TC) is defined as the union of ET and NCR.

The sample was split into training (*n* = 536), test (*n* = 67), and generalization (*n* = 44). Test and training samples were stratified based on tumor grade, operation status, scanner manufacturer, magnetic field strength, and patient sex. The generalization sample consisted of pretreatment cases, 50% of which were scanned at different sites from the training and test samples. No patient included in the training sample was included in the test or generalization sample.

### Imaging Data Acquisition and Preprocessing

The detailed acquisition parameters of the heterogeneous BraTS and TCIA scans are available elsewhere.^1,2,15^ All mpMRI scans consisted of T1-weighted (T1), T1-weighted post-contrast (T1-post), T2, and T2-FLAIR (FLAIR) images. Similar to BraTS, preprocessing consisted of co-registration to the T1-post-contrast image, resampling to isotropic 1 mm^3^ voxel resolution. Skull stripping was performed with S3 (simple skull stripping).^16^

### Reference Standard Voxelwise Annotations

The publicly available expert segmentations for the 355 BraTS 2020 cases were used as the reference standard voxelwise segmentations. Prior expert segmentation of the BraTS data set delineated 3 tumor subregions: (1) necrotic core (NCR), (2) active tumor (AT), and (3) peritumoral edematous or infiltrated tissue (ED). The whole tumor (WT) extent is defined as the union of all 3 distinct subregions (ED, AT, NCR), and the tumor core (TC) is defined as the union of AT and NCR.

For the remaining 292 cases, including the 251 postoperative cases, reference standard voxelwise segmentations were generated by 1 of 3 expert neuroradiologists or radiation oncologists (BLINDED), using ITK-SNAP.^17^ There are significant differences in the appearance of pre- and posttreatment gliomas and prior BraTS challenges^2,3^ have focused on pretreatment gliomas. Therefore, BraTS tissue labels had to be slightly redefined. With the following modifications related to the posttreatment nature of the scans, (1) resection cavities (RC) were included as their own class and included new and old resection cavities; (2) gliosis and postradiation changes manifesting as T2/FLAIR hyperintensity were also included in the ED tissue class, now called surrounding nonenhancing FLAIR hyperintensity (SNFH); and (3) smooth linear thin enhancement underlying the craniotomy or along the resection cavity was not included in the active tumor class, but any enhancing tissue that could possibly reflect tumor was included, now called enhancing tissue (ET). (4) Rather than designating a whole tumor class, a whole lesion (WL) class was defined as SNFH + ET + NCR, excluding RC.

For each of the 111 test and generalization cases, a second board-certified neuroradiologist independently segmented the cases. Dice scores were computed between the 2 segmentations to evaluate interrater variability.

### U-Net Architecture

**Figure 1.**
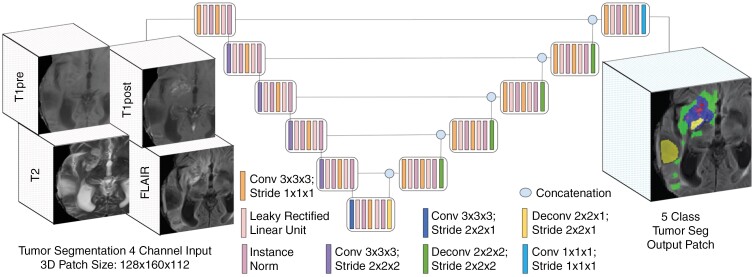
3D neural network (nnU-Net) used for diffuse gliomas segmentation with T1, T1-post, FLAIR, and T2 images used as 4 channel input and 4 class output consisting of enhancing tissue (ET; blue), necrotic core (NCR; red), surrounding nonenhancing FLAIR hyperintensity (SNFH; green), and resection cavities (RC; yellow).

We used default settings of the 3D_fullres nnU-Net^4^ a publicly available self-configuring method that automatically performs data preprocessing, network architecture configuration, and hyperparameter tuning. Batch size was 2 and 3D patch size, which was autoselected to be 128 × 128 × 128. Training was performed on a RTX 3090 GPU (CUDA version 11.2; NVIDIA Corporation; 24 GB memory) for 1000 epochs using a combination of cross entropy and Dice loss function (1:1) without any hyperparameter tuning. Input consisted of the preprocessed T1, T1-post, T2, and FLAIR images, and outputs consisted of background, ET, SNFH, NCR, and RC tissue classes. An example of the architecture, inputs, and outputs are shown in Figure 1.

### Experiments

We trained 4 different networks using a subset of the training sample. This included only the pretreatment scans (*n* = 310; Pre-Rx network), only the posttreatment scans (*n* = 226; Post-Rx network), the entire training sample (*n* = 536; Combined network), and a random subset of pre- and posttreatment scans (*n* = 226; Combined network small). The performance of these networks was evaluated on the test/generalization set (*n* = 111), which contained 87 pretreatment scans and 25 posttreatment scans.

### Performance Metrics

Tissue segmentation performance of the 4 different networks in the test/generalization set was primarily evaluated using the Dice metric (2TP/(2TP + FP + FN); TP = true positive; FP = false positive; FN = false negative).^18^ This was evaluated for cases that contained at least 0.1 mL for each respective tissue class, including SNFH, ET, NCR, RC, TC (ET + NCR), and WL (ET + NCR + SNFH), and RC + NCR. The tissue subregion Dice scores of the Combined network were compared with the Pre-Rx and Post-Rx network using paired *t* tests (two-tailed, *P < *.05). We also performed Pearson correlations between manually segmented and predicted volumes for SNFH, ET, and WL. As complementary measures, we also evaluated volume similarities and 95th percentile of the Hausdorff distance (Hausdorff^95^).

## Results

### Tumor Tissue Volumes

Tumor tissue volumes derived from manual segmentations conducted are shown in Table 1. 86% (566/647) of cases had at least 0.1 cm^3^ of ET, 69% (444/647) of patients had at least 0.1 cm^3^ of NCR, and 30% (196/647) of all cases and 78% (196/251) of posttreatment cases had a RC.

### Segmentation Performance

**Figure 2.**
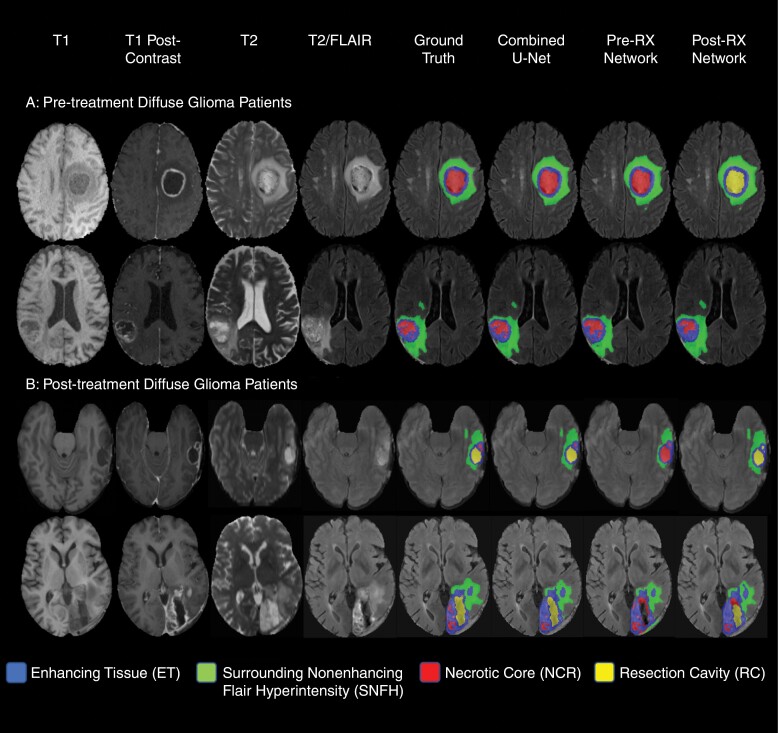
Example segmentation network predicted segmentations. Two example cases of pretreatment and posttreatment diffuse glioma MRIs with axial T2 images, T1 pre-contrast images (T1-pre), T1 post-contrast images (T1-post), FLAIR images, and example tumor tissue class segmentations overlaid on the FLAIR image (blue = enhancing tissue (ET), green = surrounding nonenhancing flair hyperintensity (SNFH), yellow = resection cavity (RC), and red = necrotic tumor core (NCR).

Mean and median (IQR 25-75) Dice scores for tissue subregions for the combined test/generalization sample with at least 0.1 cm^3^ of each respective subregion are shown in Table 2 for the Combined network, Pre-Rx network, and Post-Rx network. Results separated for the test and generalization samples shown in Supplementary Table 1. Volume similarities and Hausdorf^95^ distances for the 3 networks are found in Supplementary Table 2. Dice scores for tissue subregions for the combined test/generalization sample separated by tumor grade are shown in Supplementary Table 3. Results for the Combined network small are shown in Supplementary Table 4. Example test cases for the 3 networks are shown in Figure 2.

**Table 2.** Segmentation Performance of the Combined Network, Pre-Rx Network and Post-Rx Network on Pretreatment and Posttreatment Test/Generalization Cases

	Combined Network (*n* = 536)	Pre-Rx Network (*n* = 310)	Post-Rx Network (*n* = 226)
Pretreatment cases	Dice Mean, Median (IQR 25-75)	Dice Mean, Median (IQR 25-75)	Dice Mean, Median (IQR 25-75)
WL	0.92, 0.94 (0.92–0.95) —	0.90, 0.94 (0.92–0.95) *P* > .05	0.90, 0.92 (0.88–0.94) *P* < .05
SNFH	0.82, 0.86 (0.79–0.90) —	0.80, 0.85 (0.74–0.91) *P* = 0.02	0.79, 0.84 (0.73–0.89) *P* < .05
TC	0.86, 0.93 (0.84–0.95) —	0.87, 0.93 (0.83–0.96) *P* > .05	0.67, 0.83 (0.60–0.92) *P* < .05
ET	0.84, 0.87 (0.82–0.92) —	0.83, 0.87 (0.82–0.92) *P* > .05	0.81, 0.84 (0.80–0.89) *P* < .05
NCR	0.73, 0.82 (0.65–0.91) —	0.74, 0.83 (0.65–0.90) *P* > .05	0.38, 0.30 (0.00–0.79) *P* < .05
NCR + RC	0.74, 0.82 (0.67–0.91) —	0.74, 0.84 (0.65–0.90) *P* > .05	0.65, 0.79 (0.52–0.86) *P* < .05
RC	N/A (7% FP) —	N/A —	N/A (59% FP) —
Posttreatment cases	Dice Mean, Median (IQR 25-75)	Dice Mean, Median (IQR 25-75)	Dice Mean, Median (IQR 25-75)
WL	0.88, 0.89 (0.87–0.92) —	0.70, 0.81 (0.55–0.90) *P* < .05	0.89, 0.89 (0.87–0.92) *P* > .05
SNFH	0.82, 0.86 (0.77–0.87) —	0.69, 0.74 (0.65–0.83) *P* < .05	0.82, 0.85 (0.77–0.87) *P* > .05
TC	0.70, 0.82 (0.61–0.91) —	0.52, 0.63 (0.24–0.86) *P* < .05	0.72, 0.83 (0.62–0.89) *P* > .05
ET	0.70, 0.81 (0.64–0.89) —	0.63, 0.80 (0.51–0.86) *P* < .05	0.72, 0.83 (0.64–0.88) *P* > .05
NCR	0.38, 0.41 (0.14–0.55) —	0.20, 0.10 (0.02–0.18) *P* < .05	0.29, 0.01 (0.00–0.63) *P* > .05
NCR + RC	0.75, 0.84 (0.73–0.91) —	0.45, 0.52 (0.06–0.77) *P* < .05	0.74, 0.85 (0.69–0.90) *P* > .05
RC	0.76, 0.86 (0.73–0.91) —	0.00 (0.00–0.00) *P* < .05	0.76, 0.85 (0.74–0.91) *P* > .05

ET = enhancing tissue; NCR = necrotic core; RC = resection cavity; SNFH = surrounding nonenhancing FLAIR hyperintensity. The whole lesion (WL) extent is defined as the union of all 3 distinct subregions (ET, SNFH, and NCR), excluding resection cavity. Tumor core (TC) is defined as the union of ET and NCR.

For the pretreatment cases, the Combined network had equivalent mean Dice scores (*P* > .05 for most tissue classes) compared to the Pre-Rx network, with superior performance for SNFH (*P* = .02). The Combined network had better Dice scores compared to the Post-Rx network (*P* < .05 for all tissue classes) for pretreatment cases. For posttreatment cases, the Combined network had equivalent Dice scores to the Post-Rx network (*P* > .05 for all tissue classes), and better Dice scores than the Pre-Rx network (*P* < .05 for all tissue classes). The Combined network small demonstrated equivalent dice scores to the Pre-Rx network (*P* > .05 for all tissue classes) for pretreatment cases, except for NCR in which it was inferior (*P* < .05). The Post-Rx network was inferior to the Combined network small for pretreatment tissue volumes (*P* < .05 for most tissue classes), except for SNFH and WL in which it was equivalent (*P* > .05). The Combined network small demonstrated superior dice scores to the Pre-Rx network for all posttreatment tissue volumes (*P* < .05), with the exception of NCR which was equivalent (*P* > .05). Post-Rx network was equivalent to Combined network small in all posttreatment tissue volumes (*P* > .05).

**Figure 3.**
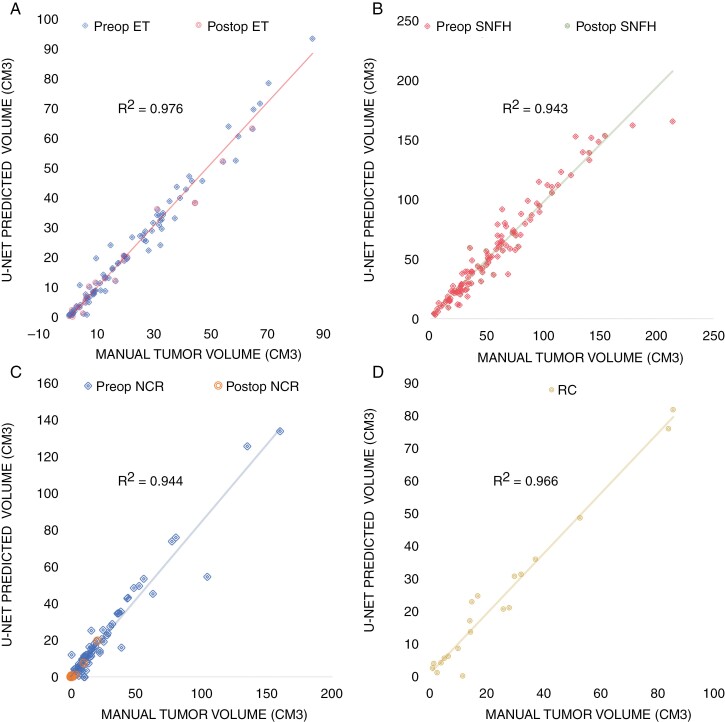
Correspondence between predicted and ground truth tumor subregion volumes. Scatter plots of manually segmented tumor subregion volumes versus U-Net predicted tumor subregion volumes for surrounding nonenhancing FLAIR hyperintensity (SNFH; A) enhancing tissue (ET; B), necrotic core (NCR; C), and resection cavity (RC; D).

The Post-Rx network had 59% false positive for resection cavities in pretreatment cases. The Combined network had only 7% false positive resection cavities in the pretreatment cases, and the Combined small network had 14% false positive resection cavities. The Pre-Rx network was unable to segment resection cavities as it was not trained on any cases with resection cavities and had relatively poor performance on posttreatment cases. There were strong correlations between segmented and predicted tissue volumes for the Combined network (Pearson *R* > 0.98 and *R*^2^ > 0.97; Figure 3).

### Interrater variability

Interrater Dice scores for different tissue classes in the test and generalization cases are shown in Table 3. Interrater Dices scores were worse than the performance of the Combined network for SNFH and TC (*P* < .05) and equivalent to the performance of the Combined network for the remaining tissue classes (*P* > .05).

**Table 3.** Dice Score Comparison Between Raters and the Combined Network

Interrater Reliability	Dice	Combined Network	Dice
	Mean, Median (IQR 25-75)		Mean, Median (IQR 25-75)
WL	0.91, 0.93 (0.88–0.96) —	WL	0.91, 0.93 (0.91–0.95) *P* > .05
SNFH	0.79, 0.85 (0.73–0.90) —	SNFH	0.82, 0.86 (0.79–0.91) *P* < .05
TC	0.78, 0.92 (0.72–0.96) —	TC	0.82, 0.92 (0.80–0.95) *P* < .05
ET	0.78, 0.87 (0.76–0.95) —	ET	0.81, 0.86 (0.80–0.91) *P* > .05
NCR	0.72, 0.88 (0.53–0.93) —	NCR	0.68, 0.81 (0.55–0.89) *P* > .05
NCR + RC	0.77, 0.88 (0.74–0.94) —	NCR + RC	0.75, 0.83 (0.71–0.91) *P* > .05
RC	0.76, 0.81 (0.69–0.91) —	RC	0.76, 0.86 (0.73–0.91) *P* > .05

ET = enhancing tissue; NCR = necrotic core; RC = resection cavity; SNFH = surrounding nonenhancing FLAIR hyperintensity. The whole lesion (WL) extent is defined as the union of all 3 distinct subregions (ET, SNFH, and NCR), excluding resection cavity. Tumor core (TC) is defined as the union of ET and NCR.

## Discussion

Assessment of glioma treatment response after surgery, chemotherapy, and radiation is challenging given complex imaging changes over time. Manual delineation of glioma volumes for treatment planning on mpMRI is time consuming and has interoperator variation. Automated segmentation of pre- and posttreatment diffuse glioma tumor subregions has significant potential to increase workflow efficiency for radiologists, radiation oncologists, and neuro-oncologists. In this study, we trained a state-of-the-art 3D U-Net neural network to segment diffuse glioma tissue subregions in pre- and posttreatment MRIs.

The vast majority of prior work has evaluated methods for automatic segmentation of diffuse glioma subregions in pretreatment patients, typically relying on the BraTS data set. Some studies began to evaluate methods for automated segmentation for posttreatment diffuse glioma subregions,^9–12^ although most without including resection cavities. Ermis et al.,^13^ in a proof-of-concept study used DenseNet to automatically segment resection cavities in 30 posttreatment diffuse glioma cases; however, Dice scores were found to underestimate RC volumes. In this study, we combined BraTS preoperative data with publicly available TCIA posttreatment data in order to train an algorithm to accurately segment glioma subregions across pre- and posttreatment MRIs, including resection cavities. Segmentation performance in the test/generalization was as good as or better than interrater reliability with excellent Dice scores (median WL > 0.94) and volume correlations (*R*^2^ > 0.97) between manually segmented and predicted tumor subregion volumes.

Overall, we found the best segmentation performance on the test/generalization data with the Combined network. Interestingly, the Combined network performed better than the Pre-Rx network for SNFH, and equivalently on the remaining tissue classes. The Combined network was equivalent on the posttreatment cases compared to the Post-Rx network for all tissue classes. The Combined network achieved a much lower false positive rate (7%) for resection cavities in pretreatment glioma patients compared to the Post-Rx network (59%). Similar trends were seen in relation to Combined network small; however, the Pre-Rx network did have superior performance for NCR in pretreatment cases (*P* < .05) and a higher RC false positive rate (14%) than the original Combined network. This highlights the importance of increasing sample sizes when segmenting tissue volumes with complex characteristics.

Overall, the findings suggest that a single combined model could be used clinically for both pre- and posttreatment gliomas rather than having separate networks for each type of scan. Not surprisingly, segmentation performance for ET and NCR was lower in posttreatment cases relative to pretreatment cases (median Dice scores of 0.87/0.81 vs 0.84/0.41 for ET/NCR in pre- vs posttreatment cases). ET was likely lower due to due to more complex enhancement patterns. NCR was likely lower due to challenges in distinguishing necrotic core from resection cavities in posttreatment gliomas.

The Combined network exhibited higher Dice scores for SNFH and TC tissue classes compared to Dice scores between two different neuroradiologists and was not significantly different for the remaining tissue classes. This suggests the Combined network was equivalent or better than radiologists’ interrater reliability, particularly for less ambiguous tissue volumes.

A major limitation was the inability to distinguish between enhancing tumor and posttreatment enhancement/pseudoprogression or between infiltrative tumor, edema, and posttreatment changes, which would require more clinical information and advanced imaging modalities such as perfusion^19^ and diffusion sequences.^20^ It also remains to be tested whether such an algorithm, when integrated into the clinical workflow could improve the accuracy and efficiency of radiology, radiation oncology, and neuro-oncology workflows.

## Conclusions

Neural networks were able to accurately segment pre- and posttreatment diffuse glioma tissue subregions including resection cavities. We found that a single network trained to segment pre- and posttreatment diffuse gliomas is as good as separate networks, which would be simpler to implement clinically. Automated volumetric quantification of diffuse glioma tissue volumes may improve response assessment in clinical trials and reduce provider burden and errors in measurement.

## Supplementary Material

vdae140_suppl_Supplementary_Tables
