# Supplementary material for: Segmentation of pre- and posttreatment diffuse glioma tissue subregions including resection cavities
Source: Neurooncol Adv. 2024 Aug 16;6(1):vdae140. doi: 10.1093/noajnl/vdae140 (PMC11407510; doi:10.1093/noajnl/vdae140)
Supplement: vdae140_suppl_Supplementary_Tables [file vdae140_suppl_supplementary_tables.docx]

**Supplementary Tables**

**Table S1.** Dice scores separated by test and generalization samples

|  | **Combined Network (n=536)** | **Pre-Rx Network (n=310)** | **Post-Rx Network (n=226)** |
| --- | --- | --- | --- |
| **Pre-Op (Test Set)** | Dice  Median (IQR 25-75) | Dice Median (IQR 25-75) | Dice  Median (IQR 25-75) |
| WL | 0.94 (0.92-0.96) | 0.94 (0.91-0.96) | 0.90 (0.80-0.93) |
| SNFH | 0.87 (0.81-0.92) | 0.88 (0.78-0.92) | 0.84 (0.74-0.89) |
| TC | 0.95 (0.90-0.96) | 0.94 (0.91-0.96) | 0.87 (0.67-0.94) |
| ET | 0.90 (0.84-0.93) | 0.90 (0.85-0.93) | 0.86 (0.81-0.91) |
| NCR | 0.88 (0.81-0.94) | 0.89 (0.82-0.94) | 0.62 (0.06-0.83) |
| NCR + RC | 0.88 (0.81-0.94) | 0.89 (0.82-0.94) | 0.84 (0.77-0.90) |
| RC | N/A | N/A | N/A |
| **Post-Op (Test Set)** | Dice  Median (IQR 25-75) | Dice Median (IQR 25-75) | Dice  Median (IQR 25-75) |
| WL | 0.87 (0.77-0.90) | 0.74 (0.61-0.82) | 0.85 (0.77-0.89) |
| SNFH | 0.85 (0.77-0.87) | 0.74 (0.65-0.83) | 0.85 (0.77-0.87) |
| TC | 0.83 (0.61-0.91) | 0.67 (0.28-0.86) | 0.83 (0.63-0.89) |
| ET | 0.82 (0.71-0.90) | 0.80 (0.56-0.86) | 0.84 (0.66-0.88) |
| NCR | 0.56 (0.33-0.76) | 0.45 (0.17-0.74) | 0.51 (0.28-0.72) |
| NCR + RC | 0.85 (0.74-0.91) | 0.55 (0.08-0.78) | 0.86 (0.73-0.91) |
| RC | 0.86 (0.73-0.91) | N/A | 0.85 (0.74-0.91) |
| **Pre-Op (Generalization Set)** | Dice  Median (IQR 25-75) | Dice Median (IQR 25-75) | Dice  Median (IQR 25-75) |
| WL | 0.94 (0.92-0.95) | 0.94 (0.92-0.95) | 0.89 (0.82-0.93) |
| SNFH | 0.85 (0.77-0.89) | 0.81 (0.73-0.88) | 0.82 (0.73-0.88) |
| TC | 0.92 (0.81-0.94) | 0.92 (0.82-0.94) | 0.79 (0.06-0.91) |
| ET | 0.84 (0.81-0.89) | 0.84 (0.80-0.87) | 0.84 (0.80-0.87) |
| NCR | 0.80 (0.64-0.84) | 0.81 (0.66-0.86) | 0.16 (0.00-0.64) |
| NCR + RC | 0.80 (0.70-0.84) | 0.81 (0.66-0.86) | 0.71 (0.47-0.82) |
| RC | N/A | N/A | N/A |
| Abbreviations: Necrotic core (NCR), enhancing tissue (ET), surrounding nonenhancing FLAIR hyperintensity (SNFH), resection cavity (RC). The whole lesion (WL) extent is defined as the union of all three distinct subregions (ET, SNFH, NCR), excluding resection cavity. Tumor core (TC) is defined as the union of ET and NCR. | | | |

**Table S2.** Volume similarity and Hausdorff95 distance metrics.

|  | **Combined Network (n=536)** | **Pre-Rx Network  (n=310)** | **Post-Rx Network  (n=226)** | |
| --- | --- | --- | --- | --- |
| **Pre-Op** | Volume Similarity  Median (IQR 25-75) | Volume Similarity  Median (IQR 25-75) | Volume Similarity  Median (IQR 25-75) | |
| WL | 0.96, 0.98 (0.96 - 0.99) | 0.94, 0.98 (0.9 5 - 0.99) | 0.94, 0.96 (0.92 - 0.98) | |
| SNFH | 0.93, 0.96 (0.91 - 0.98) | 0.90, 0.95 (0.89 - 0.97) | 0.90, 0.93 (0.88 - 0.96) | |
| TC | 0.87, 0.98 (0.90 - 0.99) | 0.88, 0.97 (0.91 - 0.99) | 0.70, 0.92 (0.64 - 0.97) | |
| ET | 0.84, 0.96 (0.89 - 0.98) | 0.82, 0.95 (0.85 - 0.98) | 0.80, 0.91 (0.82 - 0.96) | |
| NCR | 0.80, 0.93 (0.78 - 0.98) | 0.83, 0.93 (0.84 - 0.98) | 0.45, 0.37 (0.00 - 0.86) | |
| NCR + RC | 0.82, 0.93 (0.79 - 0.98) | 0.83, 0.93 (0.84 - 0.98) | 0.74, 0.87 (0.63 - 0.96) | |
| RC | N/A | N/A | 0.00 (0.00-0.00) | |
| **Post-Op** | Volume Similarity  Median (IQR 25-75) | Volume Similarity  Median (IQR 25-75) | Volume Similarity  Median (IQR 25-75) | |
| WL | 0.95, 0.97 (0.94 - 0.98) | 0.77, 0.86 (0.62- 0.95) | 0.96, 0.97 (0.96 - 0.99) | |
| SNFH | 0.91, 0.94 (0.88 - 0.97) | 0.81, 0.83 (0.74 - 0.94) | 0.92, 0.95 (0.87 - 0.98) | |
| TC | 0.79, 0.94 (0.87 - 0.98) | 0.58, 0.78 (0.21 - 0.93) | 0.80, 0.92 (0.82 - 0.96) | |
| ET | 0.78, 0.93 (0.81 - 0.98) | 0.69, 0.85 (0.56 - 0.95) | 0.81, 0.93 (0.82 - 0.97) | |
| NCR | 0.36, 0.31 (0.00 - 0.68) | 0.18, 0.00 (0.00 - 0.18) | 0.31, 0.00 (0.00 - 0.74) | |
| NCR + RC | 0.84, 0.90 (0.81 - 0.96) | 0.61, 0.83 (0.15 - 0.93) | 0.85, 0.94 (0.81 - 0.97) | |
| RC | 0.75, 0.92 (0.66 - 0.98) | 0.00 (0.00-0.00) | 0.72, 0.91 (0.59 - 0.97) | |
| **Pre-Op** | Hausdorff distance  Median (IQR 25-75) | Hausdorff distance  Median (IQR 25-75) | Hausdorff distance  Median (IQR 25-75) | |
| WL | 4.26, 2.24 (1.41 - 4.09) | 4.62, 2.24 (1.41 - 4.84) | 8.66, 5.39 (4.00 - 8.50) | |
| SNFH | 4.89, 2.44 (1.80 -5.72) | 5.35, 2.83 (2.00 - 7.16) | 6.20, 3.61 (2.24 - 8.05) | |
| TC | 4.27, 2.00 (1.41 - 5.02) | 4.13, 2.00 (1.41 - 5.00) | 10.00, 4.69 (2.45 - 9.48) | |
| ET | 3.51, 1.41 (1.00 - 2.24) | 3.37, 1.41 (1.00 - 2.83) | 4.38, 2.00 (1.41 - 3.46) | |
| NCR | 5.78, 4.24 (2.45 - 6.48) | 5.62, 3.74 (2.45 - 6.16) | 11.07, 7.71 (4.36 - 15.3) | |
| NCR + RC | 5.83, 4.24 (2.45 - 6.40) | 5.62, 3.74 (2.45 - 6.16) | 8.66, 5.39 (4.00 - 8.50) | |
| RC | N/A | N/A | N/A | |
| **Post-Op** | Hausdorff distance  Median (IQR 25-75) | Hausdorff distance  Median (IQR 25-75) | Hausdorff distance  Median (IQR 25-75) | |
| WL | 7.39, 4.12 (2.83 - 5.83) | 14.85, 8.66 (5.39 - 20.90) | 6.25, 5.15 (3.12 - 7.05) | |
| SNFH | 7.68, 4.47 (3.00 - 6.63) | 14.10, 7.28 (6.00 - 16.52) | 7.37, 4.90 (3.00 - 6.78) | |
| TC | 6.27, 3.0 (2.24 - 9.95) | 14.36, 7.64 (5.16 - 16.75) | 6.93, 3.08 (2.10 -9.53) | |
| ET | 6.30, 3.00 (2.24 - 9.95) | 8.72, 5.00 (3.61 - 12.25) | 6.94, 3.00 (2.29 - 9.54) | |
| NCR | 12.90, 12.29 (6.85 - 17.55) | 21.94, 16.73 (11.01 - 24.73) | 15.4, 9.70 (8.28 - 23.2) | |
| NCR + RC | 5.30, 4.84 (3.12 - 6.85) | 15.17, 9.11 (6.74 - 22.67) | 6.25, 5.15 (3.12 - 7.05) | |
| RC | 6.44, 4.62 (2.86 - 6.97) | N/A | 7.16, 4.35 (3.12 - 7.31) | |
| Abbreviations: Necrotic core (NCR), enhancing tissue (ET), surrounding nonenhancing FLAIR hyperintensity (SNFH), resection cavity (RC). The whole lesion (WL) extent is defined as the union of all three distinct subregions (ET, SNFH, NCR), excluding resection cavity. Tumor core (TC) is defined as the union of ET and NCR. | | | |  |

**Table S3**. Dice scores separated by High Grade (HGG) and Low Grade (LGG) Glioma

| **HGG** | **Combined Network (n=536)** | **Pre-Rx Network  (n=310)** | **Post-Rx Network  (n=226)** | |
| --- | --- | --- | --- | --- |
| **Pre-Op** | Dice  Mean, Median (IQR 25-75) | Dice  Mean, Median (IQR 25-75) | Dice  Mean, Median (IQR 25-75) | |
| WL | 0.93, 0.94 (0.93 - 0.96) | 0.93, 0.94 (0.92 - 0.94) | 0.91, 0.92 (0.90 - 0.95) | |
| SNFH | 0.85, 0.88 (0.83 - 0.92) | 0.85, 0.88 (0.83 - 0.92) | 0.82, 0.85 (0.79 - 0.89) | |
| TC | 0.92, 0.94 (0.92 - 0.96) | 0.91, 0.94 (0.92 - 0.96) | 0.84, 0.90 (0.77 - 0.94) | |
| ET | 0.86, 0.88 (0.83 - 0.92) | 0.86, 0.88 (0.83 - 0.91) | 0.83, 0.86 (0.81 - 0.90) | |
| NCR | 0.78, 0.83 (0.73 - 0.91) | 0.78, 0.84 (0.77 - 0.90) | 0.51, 0.62 (0.20 - 0.82) | |
| NCR + RC | 0.78, 0.83 (0.73 - 0.91) | 0.78, 0.84 (0.77 - 0.90) | 0.74, 0.82 (0.72 - 0.88) | |
| RC | N/A | N/A | N/A | |
| **Post-Op** | Dice  Mean, Median (IQR 25-75) | Dice  Mean, Median (IQR 25-75) | Dice  Mean, Median (IQR 25-75) | |
| WL | 0.88, 0.89 (0.87 - 0.92) | 0.70, 0.81 (0.55 - 0.90) | 0.89, 0.89 (0.87-0.92) | |
| SNFH | 0.81, 0.85 (0.77 - 0.87) | 0.69, 0.74 (0.65 - 0.83) | 0.82, 0.85 (0.77-0.87) | |
| TC | 0.70, 0.82 (0.61 - 0.91) | 0.52, 0.63 (0.24-0.86) | 0.72, 0.83 (0.62-0.89) | |
| ET | 0.70, 0.81 (0.64-0.89) | 0.63, 0.80 (0.51-0.86) | 0.72, 0.83 (0.64-0.88) | |
| NCR | 0.38, 0.41 (0.13 - 0.55) | 0.20, 0.10 (0.02-0.20) | 0.29, 0.02 (0.00-0.63) | |
| NCR + RC | 0.75, 0.84 (0.72-0.91) | 0.45, 0.52 (0.06 - 0.76) | 0.74, 0.85 (0.69-0.90) | |
| RC | 0.76, 0.86 (0.73 - 0.91) | N/A | 0.76, 0.85 (0.74-0.91) | |
| **LGG** |  |  |  | |
| **Pre-Op** | Dice  Mean, Median (IQR 25-75) | Dice  Mean, Median (IQR 25-75) | Dice  Mean, Median (IQR 25-75) | |
| WL | 0.84, 0.92 (0.91-0.94) | 0.83, 0.92 (0.91-0.94) | 0.89, 0.92 (0.86-0.94) | |
| SNFH | 0.65, 0.75 (0.54-0.86) | 0.67, 0.73 (0.67-0.77) | 0.72, 0.74 (0.59-0.84) | |
| TC | 0.66, 0.75 (0.61-0.88) | 0.71, 0.80 (0.69-0.90) | 0.13, 0.01 (0.00-0.13) | |
| ET | 0.65, 0.77 (0.48-0.85) | 0.61, 0.75 (0.32-0.86) | 0.58, 0.72 (0.32-0.81) | |
| NCR | 0.57, 0.72 (0.28-0.86) | 0.64, 0.75 (0.51-0.89) | 0.01. 0.00 (0.00-0.00) | |
| NCR + RC | 0.65, 0.75 (0.54-0.86) | 0.64, 0.75 (0.51-0.89) | 0.37, 0.42 (0.03-0.60) | |
| RC | N/A | N/A | N/A (% FP) | |
| Abbreviations: Necrotic core (NCR), enhancing tissue (ET), surrounding nonenhancing FLAIR hyperintensity (SNFH), resection cavity (RC). The whole lesion (WL) extent is defined as the union of all three distinct subregions (ET, SNFH, NCR), excluding resection cavity. Tumor core (TC) is defined as the union of ET and NCR. | | | |  |

**Table S4**. Dice scores for subsampled training data for the Combined Network Small

|  | **Combined Network Small (n=226)** | **Pre-Rx Network (n=310)** | **Post-Rx Network (n=226) -148** |
| --- | --- | --- | --- |
| **Pre-treatment cases** | Dice | Dice | Dice |
|  | Mean, Median (IQR 25-75) | Mean, Median (IQR 25-75) | Mean, Median (IQR 25-75) |
| WL | 0.91, 0.94 (0.92 - 0.95) | 0.90, 0.94 (0.92-0.95) | 0.90, 0.92 (0.88-0.94) |
|  | - | P > 0.05 | P > 0.05 |
| SNFH | 0.80, 0.84 (0.76 - 0.90) | 0.80, 0.85 (0.74-0.91) | 0.79, 0.84 (0.73-0.89) |
|  | - | P > 0.05 | P > 0.05 |
| TC | 0.83, 0.93 (0.81 - 0.95) | 0.87, 0.93 (0.83-0.96) | 0.67, 0.83 (0.60-0.92) |
|  | - | P > 0.05 | P < 0.05 |
| ET | 0.83, 0.86 (0.81 - 0.91) | 0.83, 0.87 (0.82-0.92) | 0.81, 0.85 (0.80-0.89) |
|  | - | P > 0.05 | P < 0,05 |
| NCR | 0.70, 0.82 (0.58 - 0.89) | 0.74, 0.83 (0.65-0.90) | 0.38, 0.30 (0.00-0.79) |
|  | - | P < 0.05 | P < 0.05 |
| NCR + RC | 0.72, 0.82 (0.65 - 0.90) | 0.74, 0.84 (0.65-0.90) | 0.65, 0.79 (0.52-0.86) |
|  | - | P = 0.05 | P < 0.05 |
| RC | N/A (14% FP) | N/A | N/A (59% FP) |
|  |  | - | - |
| **Post-treatment cases** | Dice | Dice | Dice |
|  | Mean, Median (IQR 25-75) | Mean, Median (IQR 25-75) | Mean, Median (IQR 25-75) |
| WL | 0.88, 0.90 (0.87 - 0.92) | 0.70, 0.81 (0.55-0.90) | 0.89, 0.89 (0.87-0.92) |
|  | - | P < 0.05 | P> 0.05 |
| SNFH | 0.81, 0.85 (0.76 – 0.88) | 0.69, 0.74 (0.65-0.83) | 0.82, 0.85 (0.77-0.87) |
|  | - | P < 0.05 | P > 0.05 |
| TC | 0.69, 0.81 (0.61 - 0.89) | 0.52, 0.63 (0.24-0.86) | 0.72, 0.83 (0.62-0.89) |
|  | - | P < 0.05 | P > 0.05 |
| ET | 0.69, 0.82 (0.62-0.89) | 0.63, 0.80 (0.51-0.86) | 0.72, 0.83 (0.64-0.88) |
|  | - | P < 0.05 | P > 0.05 |
| NCR | 0.29, 0.16 (0.09 - 0.51) | 0.20, 0.10 (0.02-0.18) | 0.29, 0.01 (0.00-0.63) |
|  | - | P > 0.05 | P > 0.05 |
| NCR + RC | 0.73, 0.82 (0.70 – 0.90) | 0.45, 0.52 (0.06-0.77) | 0.74, 0.85 (0.69-0.90) |
|  | - | P < 0.05 | P > 0.05 |
| RC | 0.70, 0.82 (0.68 - 0.90) | 0.00 (0.00-0.00) | 0.76, 0.85 (0.74-0.91) |
|  | - | P < 0.05 | P > 0.05 |
| Abbreviations: Necrotic core (NCR), enhancing tissue (ET), surrounding nonenhancing FLAIR hyperintensity (SNFH), resection cavity (RC). The whole lesion (WL) extent is defined as the union of all three distinct subregions (ET, SNFH, NCR), excluding resection cavity. Tumor core (TC) is defined as the union of ET and NCR. | | | |
